# Food and nutrient intake of school-aged children in Lebanon and their adherence to dietary guidelines and recommendations

**DOI:** 10.1186/s12889-022-13186-w

**Published:** 2022-05-10

**Authors:** Lara Nasreddine, Nahla Hwalla, Fatima Al Zahraa Chokor, Farah Naja, Lynda O’Neill, Lamis Jomaa

**Affiliations:** 1 Department of Nutrition and Food Sciences, Faculty of Agricultural and Food Sciences, American University of Beirut, Beirut, 11-0236 Lebanon; 2 Department of Clinical Nutrition and Dietetics, College of Health Sciences, Research Institute of Medical & Health Sciences (RIMHS), University of Sharjah, 27272 Sharjah, United Arab Emirates; 3 Faculty of Agricultural and Food Sciences, American University of Beirut, Riad El-Solh, P.O. Box 11-0236, Beirut, 1107-2020 Lebanon; 4 Nestlé Institute of Health Sciences, Nestlé Research, Société Des Produits Nestlé S.A, Vers-Chez-Les-Blancs, 1000 Lausanne 26, Lausanne, Switzerland; 5 Department of Human Sciences, College of Health and Sciences, North Carolina Central University, Durham, NC 27707 USA

**Keywords:** Nutrients, Intake, Inadequacies, Dietary guidelines, Children, Adolescents, Lebanon

## Abstract

**Background:**

Lebanon, an Eastern Mediterranean country, is witnessing a remarkable nutrition transition, and the diets of school-aged children may be amongst those most affected. However, limited studies have examined the food consumption patterns and nutrient adequacy in this age group.

**Objectives:**

The present study aimed to evaluate the dietary intakes of school-aged children in Lebanon and assess their adherence to nutrition guidelines and recommendations.

**Methods:**

This study used data for 4–13 y-old children (*n* = 711) from a national cross-sectional survey conducted in 2014–2015 on a representative sample of Lebanese households with children. Dietary intake was assessed using single 24-h recall method. Estimated food group and nutrient intakes were compared to dietary recommendations and age-specific dietary reference intakes (DRI), including Estimated Average Requirements (EAR) and Acceptable Macronutrient Distribution Range (AMDR). Food group, energy, macro- and micro-nutrient intakes were presented for all children in the sample and stratified by age (4–8 y and 9–13 y) and sex.

**Results:**

Mean energy intake of 4–13-year-old children was 1804 kcal/d. Almost half of the energy was provided by carbohydrates while 12% of children had protein intakes below EAR. Approximately three-quarters of children (4–13 y) exceeded the AMDR for total fat and saturated fats, and a similar proportion over consumed added sugars. The main sources of energy intake (EI) among children were the sweets, sweetened beverages and desserts followed by grains and mixed dishes. No significant differences were noted in %EI from different food groups, by sex, in either age groups. The highest adherence of children to food group recommendations was observed for the grains’ food group (47.2–54.4%EI), while the lowest adherence was found for vegetables (3.1–14.1%EI). A high prevalence of vitamin and mineral inadequacies was noted amongst 4–13 y old children for key micronutrients, including vitamin D (99%), calcium (81%), and vitamin A (69.5%). Risk of inadequate micronutrient intakes was significantly increased among the older age group (*p*-value < 0.05).

**Conclusion:**

Nutrient intakes of school-aged children in Lebanon reflect suboptimal nutrition. Educational and public health interventions are needed to promote healthier diets among children and prevent micronutrient deficiencies during this critical phase.

**Supplementary Information:**

The online version contains supplementary material available at 10.1186/s12889-022-13186-w.

## Introduction

Proper nutrition during childhood is an integral component for healthy growth and development and to prevent risk of diseases later in life [1]. School-aged children are particularly prone to inadequate dietary behaviors that affect their nutritional status and increase their risk of excessive weight gain and associated comorbidities. According to the World Health Organization (WHO), over 340 million children and adolescents aged 5–19 were overweight or obese in 2016 worldwide; which reflects a four-fold increase in the prevalence of pediatric overweight and obesity in 2016 compared to few decades ago (18% in 2016 compared to 4% in 1975) [2]. Pediatric obesity remains a serious public health challenge for children globally, particularly in low to middle income countries (LMICs) that are undergoing a rapid nutrition transition. This transition is characterized by the increased urbanization and modernization accompanied by the adoption of unhealthy dietary behaviors and sedentary lifestyles; factors that have been contributing to the dramatic increase in overweight and obesity among all age groups, especially among children [3–5].

In many countries undergoing nutrition transition, overweight and obesity are co-existing along with undernutrition and ‘hidden hunger’ (micronutrient deficiencies). The overlap of overnutrition and micronutrient deficiencies among school -aged children, referred to as the ‘double burden of malnutrition’, particularly in LMICs, can have serious detrimental effects in the short and long-term. Studies have shown that excessive weight gain contributes to metabolic adaptations in sugar and lipid profiles, which in turn are associated with early onset of diseases among children, such as type 2 diabetes mellitus, hypertension, and cardiovascular diseases [5, 6]. In addition, overweight and obesity during childhood has been strongly associated with adult obesity and non-communicable diseases later in life [7–10]. Concurrently, micronutrient deficiencies can have serious adverse effects on children’s physical growth [11, 12], psychosocial development [13, 14] and academic performance [13, 15] as well as their economic productivity [14, 16] during adulthood. Poor nutrition is also of particular concern for girls as their risk of malnutrition during childhood can span a life cycle and across generations contributing to the ‘intergenerational effects of malnutrition’[15, 17]. Thus, for girls, adequate nutrition is imperative at all stages of their growth and development to ensure their health and that of their future offspring.

The Eastern Mediterranean Region (EMR) is one of the regions witnessing the rapid nutrition transition along with an increased burden of diseases and malnutrition. A recent review on the nutritional status and dietary intake of children in several EMR countries reported the multiple burden of malnutrition including alarming overweight and obesity rates ranging between (11.5% -34.9% and 4.7 and 23%, respectively) among school-aged children(5–12 years) in addition to key micronutrient deficiencies [18–20]. The nutrition transition accompanying the environmental and societal changes across countries in the EMR over the past few decades have been shifting the diets of children from consuming diverse and healthy diets that are rich in fruits, vegetables, whole grains and legumes towards more processed, energy-dense and nutrient-poor foods [21, 22]. Such dietary shifts towards less traditional dietary patterns and the adoption of more Westernized food consumption behaviors that are high in energy, animal-source fats, added sugars, and salt are contributing to the excessive weight gain (overnutrition) among children, while masking persistent micronutrient deficiencies, including iron, vitamin A and vitamin D [18, 23–25].

Lebanon is a middle-income country in the EMR with alarmingly increasing overweight and obesity rates reaching 21.2% and 10.9%, respectively, amongst 6–19 year-old-children[26]. In parallel, a worrisome shift in the food consumption behaviors of Lebanese children and youth has been documented in the past two decades with the increased adoption of the Westernized dietary pattern and lower adherence to the more traditional Lebanese Mediterranean diet [27–29]. These dietary shifts were also associated with increased risk of overweight and obesity and increased risk of diabetes and metabolic syndrome among children and adolescents [26, 27, 30]. According to Naja et al., the temporal trends in food consumption patterns of Lebanese children witnessed between 1995 and 2009 provide worrisome projections with only 14–20% of Lebanese adolescents expected to remain adherent to the Mediterranean dietary pattern by 2030 [29].

Using dietary data from a national cross-sectional survey conducted in 2014–2015 on a representative sample of Lebanese households with children and adolescents, the present study aimed to characterize the dietary intake of school-aged children in Lebanon. More specifically, the study aimed to: 1) evaluate the energy, macro- and micronutrient intakes of Lebanese children (4–13 years old); 2) explore the consumption of children for specific food groups using the American Heart Association/American Academy of Pediatrics (AHA/AAP) recommendations for healthy eating patterns in children; and 3) assess the adherence of children in the study sample to US-based and international dietary guidelines and recommendations (including AHA/AAP, the US-dietary reference intakes (DRIs), and the World Health Organization guidelines), while considering age and sex-based differentials.

## Materials and methods

### Study design

This study is based on data collected by a national cross-sectional survey conducted in Lebanon in 2014–2015 entitled “*Lebanese Food and Nutrition Security Survey”* (L-FANUS). The survey included a representative sample of Lebanese households with 4–18 year old children and adolescents [31]. The sample size calculation for the original survey considered the primary objectives of the study, including the prevalence of obesity among children using previous estimates from the country (10.9% obesity prevalence amongst 6–18 years in 2009) [26]. Accordingly, a minimum sample of 1200 children were needed with a 1.8% margin of error and a 95% Confidence Interval (CI) considering incomplete data. The primary sampling unit for the survey was the household. The selection of households was based on a stratified cluster sampling strategy: the strata were the six governorates of Lebanon and the clusters were selected further at the level of districts. In each district, the selection of households was based on a probability proportional to size approach, with a higher number of participating households being drawn from more populous districts; the selection of households in districts was conducted using systematic sampling.

For a household to be eligible, it should include a Lebanese mother and her child between the ages of 4 and 18 years, both mother and child should be present at the time of the interview and not have medical conditions or chronic diseases that may affect growth or nutritional status. A total of 4076 households were approached and 3147 agreed to participate (response rate 77%). Of these, 1221 households met the eligibility criteria, of which 1209 completed the interview. Details about the original survey protocol are published elsewhere [31].

Considering the exploratory nature of the present study and given the lack of available studies in this context that would have been informative in setting an estimated prevalence of adequacy for the sample size calculation [18], a conservative 50 percent estimate was adopted as it would lead to the largest sample size estimations [32]. Thus, a total sample of 583 children was needed considering a ± 4% margin of error and a 95% confidence interval [33]. For the present study, and in line with the protocol of the Kids Nutrition and Health Study (KNHS) that was adopted in reporting and analysis [34], data pertinent to school-aged children between 4 to 13 years were considered for analysis (*n* = 711).

### Ethical considerations

The survey was performed according to the guidelines laid down in the Declaration of Helsinki and was approved by the Institutional Review Board of the American University of Beirut (NUT.LJ.03). Written informed consent was obtained from mothers prior to enrollment in the study and assent was obtained from children above 6 years of age.

### Data collection

Data was obtained through face-to-face interviews. For children aged less than 10 years, the interview was conducted with the mother, as a proxy, in the presence of the child. For children aged 10 years or above, the interview was conducted directly with the child, in the presence of the mother for assistance. Interviews were held in the household setting and lasted for approximately one hour. Trained nutritionists collected data, using age-specific multi-component questionnaires covering information on demographic, socioeconomic, anthropometric, and dietary intakes. Demographic characteristics consisted of the following: sex of the child, age of the child (years), and governorate. Socioeconomic status (SES) indicators included mother’s and father’s educational levels (less than elementary; elementary to secondary; or university degree) as well as maternal and paternal employment status (employed vs not employed). Another SES indicator was the household’s monthly income that was categorized as such: < 1,000,000 Lebanese pounds (LBP), equivalent to 666 USD, a value slightly higher than the minimum wage salary in Lebanon of 675,000 LBP (450 USD) with 1500 LBP per 1 USD conversion rate at the time of data collection; 1,000,000–2,000,000 LBP (666 – 1,333 USD); or > 2,000,000 LBP (> 1,333 USD). In addition, crowding index, another SES indicator, was calculated based on the number of rooms and number of individuals living in the household).

### Anthropometric assessment

Anthropometric characteristics including weight and height were measured for all participating children. Height measurements were obtained without shoes, using a stadiometer, and body weight was measured to the nearest 0.1 kg with the participant in light indoor clothing and with bare feet or stockings, using a standard clinical scale (Seca 11,770). All measurements of weight and height were taken twice, and the average values were adopted. BMI was calculated as the ratio of weight (kilograms) to the square of height (meters) [35].

### Dietary intake assessment

Dietary assessment was based on single multiple pass 24-h recalls (24-HRs), which were conducted using the United States Department of Agriculture (USDA) multiple pass five-step approach: 1) quick food list recall, 2) forgotten food list probe, 3) time and occasion at which foods were consumed, 4) detailed overall cycle and 5) final probe review of the foods consumed [36]. While collecting the dietary data, specific attention was made to obtain information about foods consumed at school. For young children, and in case another caretaker shared the responsibility of feeding the child, the mother directly consulted with him/her for further information pertinent to the dietary interview.

The Nutritionist Pro software (version 5.1.0, 2014, First Data Bank, Nutritionist Pro, Axxya Systems, San Bruno, CA, USA) was used for the analysis of dietary intake data. Within the Nutritionist Pro, the USDA database was selected for analysis. For composite, mixed and traditional Lebanese dishes, standardized recipes were added to the Nutritionist Pro software using local food composition databases [37]. This allowed for the estimation of energy (kcal), macro-, and micro-nutrient daily intakes. For added sugars that were missing for several food items from the USDA database, the authors calculated sugar intake based on the 10-step methodology proposed by Kibblewhite et al. (2017) [38, 39], which consists of a modification of the method of Louie et al.2015 [38].

Food items, as consumed, were categorized into 10 food groups based on similarity in nutrient profile and culinary use. The food groups included grain and grain products; fruits; vegetables; milk and milk products; meats and other protein sources; mixed dishes; savory snacks; sweets, sweetened beverages and desserts; fats and oils; and condiment and sauces. These food groups were in line with the KNHS protocol, which is a dietary intake survey of large-scale cross-sectional samples of children designed to investigate nutrient intakes, eating patterns, food sources of nutrients, and key behaviors related to energy intake and expenditure in different countries around the world [40, 41].

To allow for comparison with the American Heart Association/American Academy of Pediatrics (AHA/AAP) recommendations for healthy eating patterns in children [42, 43], all recipes were further disaggregated into their individual ingredients. Individual food items were then grouped into five main groups (Milk/Dairy; Lean Meats/Beans; Fruits; Vegetables; Grains), and intakes of these food groups were compared with the AHA/AAP recommended number of servings from each food group (by child’s sex and age group) [see additional file 3 for more details] [42, 43]. The grains food group included refined and whole grain foods such as whole wheat bread, oat bran bread, bran flakes cereals, dry oat bran, and durum wheat. As for the lean meats and beans group, it included lean meats, beans, legumes, fish and eggs. The high fat meats were excluded from this group if it contained > 10 g total fat in each 100 g of meat, as per U.S. Department of Agriculture guidelines [44].

### Data analysis

Analysis was conducted using Stata software (version 16.0). All analyses were stratified by two age groups (4 to 8 years and 9 to 13 years), in line with the US DRIs and the KNHS protocol.

Sociodemographic and anthropometric characteristics were described using frequencies and percentages for categorical variables. Anthropometric characteristics were interpreted using the WHO-2006 criteria for children under-five years [45] and WHO-2007 criteria for children above-five years [46]. Food sources of energy (Kcal/capita/day) and percent contribution to energy intake (%EI) were calculated. Normality of these continuous variables were tested using the Shapiro–Wilk test and skewness tests using the “swilk” and “sktest” commands on Stata. Variables that had a non-normal distribution (p-value < 0.05) were expressed as medians, 25th percentile and 75th percentile (P25, P75). Differences in the intakes of food groups by age group were explored using the non-parametric Mann–Whitney U test. Percentage of adherence to the AHA/AAP dietary recommendations were also assessed in the present study, and differences between age groups were evaluated using chi-square tests.

The distribution of macro- and micro-nutrient intakes were reported as means and SE, medians, 25^th^ percentile, and 75^th^ percentile. The percentage of kilocalories from macronutrients were also calculated and presented as % energy intake (EI). Estimated nutrient intakes were compared to age-specific DRIs established by the Institute of Medicine (IOM), including the Estimated Average Requirement (EAR), Adequate Intake (AI) [47], and the Acceptable Macronutrient Distribution Range (AMDR). Sodium and potassium DRIs were based on the updated National Academies recommendations [48]. For nutrients with an EAR, the proportions of children with intakes less than the EAR (referring to inadequate intakes) was calculated. AI was used for nutrients that do not have an EAR value, and the percentage of children consuming greater than or equal to the AI was calculated. AI is “a recommended average daily nutrient intake level based on observed or experimentally determined approximations or estimates of nutrient intake by a group (or groups) of apparently healthy people that are assumed to be adequate” [49]. Thus, an average mean intake at or above the AI indicates that the prevalence of inadequacy is probably low [49]. If a group’s mean intake is below the AI, then intakes may need to increase, but it is not possible to precisely quantify the prevalence of nutrient inadequacy[49]. The proportions of children with intakes outside the upper or lower bounds of the AMDR for fat, protein and carbohydrate were also examined. WHO upper limit for saturated fat (8% of EI) and the AHA recommendation for added sugars (no more than 25 g per day) [50] were used to determine percentages exceeding this upper limits [51]. Differences in percentages of DRI compliance by age groups and between boys and girls within each age group were tested using chi-square tests, and differences were considered statistically significant at *p*-value < 0.05.

## Results

### Sociodemographic and anthropometric characteristics

Table 1 shows the sociodemographic and anthropometric characteristics of the study sample, which consisted of 771 children equally distributed by sex (51.9% boys and 48.1% girls), and between 4–8-year-old (50.6%) and 9–13-year-old age groups (49.4%). The majority of children were from Mount Lebanon (about 39% for each age group). Only 20.6% of children had a monthly family income greater than 2,000,000 LBP (equivalent to 1,333 USD) and almost 30% of the households had a crowding index above 2 (indicating a lower socioeconomic status). The majority of children's mothers and fathers had an educational level ranging between elementary and secondary school. While almost all of the children's fathers were employed (96.6%), around three-quarters of mothers did not work at the time of the survey (74.8%). No significant differences were observed between 4–8-year-old and 9–13-year-old children, except for mother’s education, whereby the proportion of mothers with a college degree was higher among the 4 to 8 year-old children (p-value = 0.037). The prevalence of stunting was estimated at 3.9% in the total sample, with significant differences between age groups (5.7% among 4–8-year-old children compared to 2.1% among 9–13-year-old children). Approximately a quarter of participating children (23.7%) were at risk of overweight or overweight and the prevalence of obesity was 20.2% in the overall study sample. No significant differences in BMI status were observed between the two age groups.

**Table 1:** Socio-demographic and anthropometric characteristics of Lebanese children aged 4 to 13 years, by age group

Age group (years)	4 to 13		4 to 8		9 to 13		*P*-value
	**n**	**%**	**n**	**%**	**n**	**%**	
	771	100	390	50.6	381	49.4	
**Sociodemographic Characteristics**							
**Sex**							
Boys	400	51.9	196	50.3	204	53.5	0.361
Girls	371	48.1	194	49.7	177	46.5	
**Governorate**							
Beirut	92	11.9	54	13.8	38	10.0	0.313
Mount Lebanon	302	39.2	154	39.5	148	38.8	
North Lebanon	148	19.2	68	17.4	80	21.0	
South and Nabatieh	130	16.9	61	15.6	69	18.1	
Bekaa	99	12.8	53	13.6	46	12.1	
**Household monthly income (Lebanese pounds)** ^a^							
< 1,000,000	312	40.5	153	39.2	159	41.7	0.421
1,000,000–2,000,000	289	37.5	156	40.0	133	34.9	
> 2,000–000	159	20.6	77	19.7	82	21.5	
Do not know / Refused to answer	11	1.4	4	1.0	7	1.8	
**Crowding Index**							
< 2 persons/room	539	70.1	275	70.9	264	69.3	0.631
≥ 2 persons/room	230	29.9	113	29.1	117	30.7	
**Education of mother**							
Less than elementary ^b^	14	1.8	3	0.8	11	2.9	**0.037**
Elementary-secondary ^c^	586	76.0	292	74.9	294	77.2	
College	171	22.2	95	24.4	76	19.9	
**Education of father**							
Less than elementary ^b^	25	3.3	13	3.3	12	3.2	0.933
Elementary-secondary ^c^	632	82.5	319	82.0	313	83.0	
College	109	14.2	57	14.6	52	13.8	
**Mother employed**							
Yes	194	25.2	106	27.2	88	23.1	0.185
No	576	74.8	283	72.8	293	76.9	
**Father employed**							
Yes	739	96.6	376	96.7	363	96.5	0.930
No	26	3.4	13	3.3	13	3.5	
**Anthropometric Characteristics**							
**Height for age** ^d^							
Stunted (HAZ < -2SD)	30	3.9	22	5.7	8	2.1	**0.011**
Not stunted	740	96.1	367	94.3	373	97.9	
**BMI Status** ^e^							
Wasted (BAZ < -2SD)	6	0.8	3	0.8	3	0.8	0.238
Normal	426	55.4	229	59.0	197	51.7	
At risk of overweight/Overweight ^f^(BAZ > + 1SD)	182	23.7	85	21.9	97	25.5	
Obese (BAZ > + 2SD)	155	20.2	71	18.3	84	22.0	

^a^ USD = 1, 500 Lebanese pounds (indicated rate was at the time of data collection – 2014–2015

^b^ Less than elementary includes being illiterate, not attending school, or being able to read and write only

^c^ Elementary to secondary includes primary school, intermediate school, high school, or technical diploma

^d^ Stunted if Height-for-age z-score (HAZ) < -2, not stunted if HAZ ≥ -2

^e^ Anthropometric measurements (including BMI-for-age z-score (BAZ)) of children were categorized based

on the World Health Organization (WHO) classification

and cut-off values[45, 46]

^f^ Among 4–5 years children (*n* = 72), 17 were at risk of overweight

### Food group intake

**Fig. 1:**
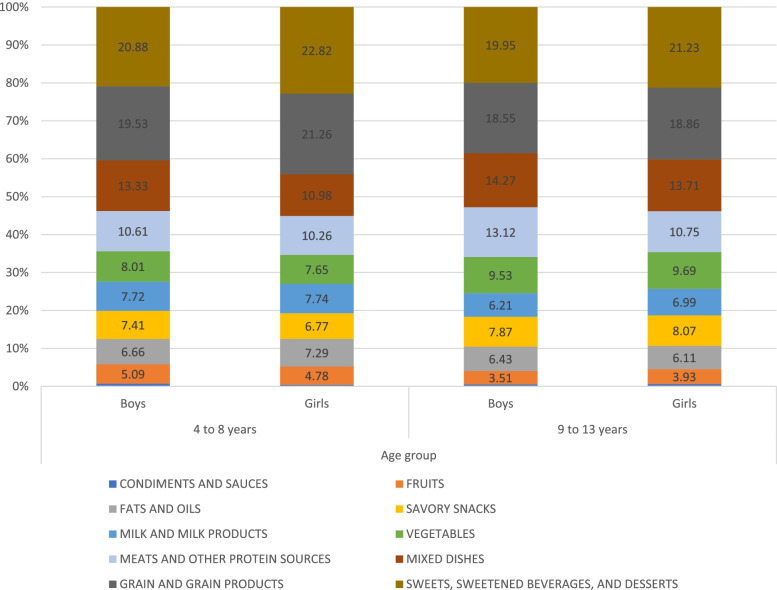
Mean percent of energy from major food groups in Lebanese children, by age and sex

Food items, as consumed, were categorized into 10 food groups. Figure 1 shows the mean intakes of food groups as percent of energy intake (%EI) for children aged 4 to 8 years and 9 to 13 years, by gender. The major source of dietary energy for children in both age groups came from the sweets, sweetened beverages, and desserts group. For children aged 4–8 years, 20.9% and 22.8% of EI for boys and girls, respectively, came from the sweets, sweetened beverages and desserts group; and for children aged 9 to 13 years, this food group represented 19.9% and 21.2% of EI for boys and girls, respectively. Other important contributors to EI in children included grains/grain products and mixed dishes, with the latter being slightly higher among boys compared to girls (13.3% versus 10.9% among children aged 4 to 8 years and 14.3% versus 13.7% among children aged 9 to 13 years). Among children aged 9 to 13 years, grains contributed to around 19% of energy intake, followed by mixed dishes (around 14%), meats (13.1% in boys versus 10.8% in girls), and vegetables (9.5% in boys versus 9.7% in girls), with no significant sex differences for any food groups observed in this age group. The median and mean intakes for the 10 food groups were presented (in grams, kilocalories, and percent EI) amongst Lebanese children aged 4 to 13 years per capita, and by age groups [see Additional files 1 and 2].

### Food group adherence

**Fig. 2:**
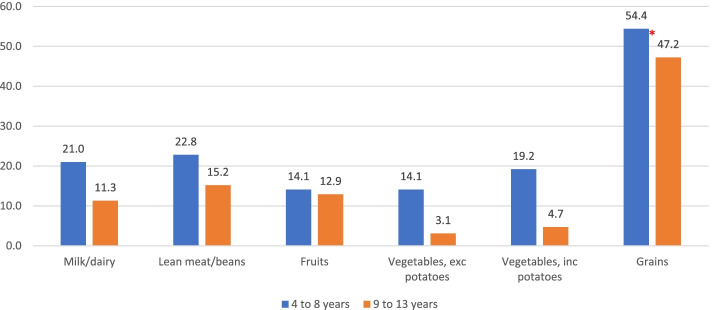
Proportion of 4–13 years old children adhering to food group recommendations, by age group† †Adherence assessment was based on the recommended servings for the various food groups by age and sex as per AHA/AAP [42, 43]. Significant differences between the age groups in the proportion of children adhering to food group recommendations indicated as * *p*-value < 0.05 & **p < 0.001

The intake level of children in the present study from different food groups was also compared to dietary recommendations set by AHA/AAP [8, 9] (see Fig. 2 and additional file 3). In children aged 4 to 13 years, the highest adherence was observed for the grains and grain products food group (47.2–54.4%), while the lowest adherence was found for vegetables (3.1–14.1%). When exploring further the grains and grain products food group in the study sample (4–13 years old), only 46 children (5.9%) reported the consumption of whole grain food items (data not shown). In addition, less than a quarter of the children in both age groups were adherent to lean meat and beans food group (22.8% in 4 to 8 years old versus 15.2% in the 9 to 13 years age group, *p* = 0.007). Significant differences in adherence to milk and dairy intake recommendations were also noted between the two age groups: 21.0% of children aged 4–8 years were adherent compared to only 11.3% of older children aged 9–13 years (*p* < 0.001). Children aged 9–13 years old also had significantly lower intake of grains compared to 4–8-year-old children (47.2% vs 54.4%, *p* = 0.048) and lower vegetables consumption (see Fig. 2, vegetables excluding potatoes, and vegetables including potations, all *p*-values < 0.001).

### Energy and nutrient intakes

Means and medians of energy and nutrient intakes for the study sample and DRI compliance for all children and by age group are shown in Table 2. Mean energy intake of the study sample was 1804 kcal. Almost half of the energy was provided by carbohydrates and 39% EI was from fat, while 12% of EI was from protein. The mean intakes of total sugars and dietary fiber were 77.6 and 14.2 g/day, respectively.

**Table 2:** Macro- and micronutrient intakes for children aged 4 to 13 years in the study sample

Nutrient	Nutrient intake		DRI^1^ (AI/EAR/AMDR)		% DRI compliance					
	**Total sample (*n* = 771)**		**4-8y**	**9-13y**	**4 to 13 years (*n* = 771)**		**4 to 8 years (*n* = 390)**		**9 to 13 years (*n* = 381)**	
	**Mean ± SE**	**Median (P25, P75)**			** < EAR/AMDR**	** > AI/AMDR**	** < EAR/AMDR**	** > AI/AMDR**	** < EAR/AMDR**	** > AI/AMDR**
**Energy (kcal/d)**	1804.1 ± 27.3	1692.0 (1268.1, 2240.4)	–	–	–	–	–	–	–	–
**Macronutrients**										
Total Fat (g/d)	80.3 ± 1.5	73.6 (50.5, 103.3)	–	–	–	–	–	–	–	–
Saturated fat (g/d)	21.5 ± 0.5	18.8 (11.6, 27.8)	–	–	–	–	–	–	–	–
Carbohydrate (g/d)	222.2 ± 3.6	210.2 (148.4, 274.4)	**100**	**100**	8.0	–	10.3 ^a^	–	5.8 ^b^	–
Total Sugar (g/d)	77.6 ± 1.8	69.3 (42.2, 100.2)	**–**	–	–	–	–	–	–	–
Added Sugars (g/d)	51.2 ± 1.6	41.7 (21.2, 71.4)	25 ^a^	25 ^a^	29.1	70.9	33.6 ^a^	66.4 ^a^	24.4 ^b^	75.6 ^b^
Dietary fiber (g/d)	14.2 ± 0.3	12.3 (8.3, 18.4)	25	31 (B)-26 (G)	–	7.7	–	7.2	–	8.1
Protein (g/d)	53.6 ± 1.0	48.4 (35.1, 67.0)	**0.76 g/Kg**	**0.76 g/Kg**	12.1	–	6.4 ^c^	–	17.9 ^d^	–
Total Fat (% of EI)	39.2 ± 0.3	39.7 (34.3, 45.1)	*25–35%*	*25–35%*	7.1	71.3	7.2	69.7	7.1	73
Saturated fat (% of EI)	10.6 ± 0.2	9.9 (7.1, 13.5)	< *8%†*	< *8%†*	–	67.8	–	68.2	–	67.5
Carbohydrate (% of EI)	49.8 ± 0.3	49.5 (43.5, 55.3)	*45–65%*	*45–65%*	30.2	6.1	26.7 ^a^	7.4 ^a^	33.9 ^b^	4.7 ^b^
Total Sugar (% of EI)	17.4 ± 0.3	16.6 (11.4, 22.3)	*–*	*–*	–	–	–	–	–	–
Added Sugar (% of EI)	11.2 ± 0.3	10.0 (5.9, 15.7)	*–*	*–*	–	–	–	–	–	–
Protein (% of EI)	12.2 ± 0.2	11.5 (9.1, 14.3)	*10–30%*	*10–30%*	33.9	0.9	32.8	0.8	34.9	1.0
**Micronutrients**										
Vitamin C (mg/d)	70.5 ± 2.6	47.8 (23.8, 93.7)	**22**	**39**	31.7	–	25.1 ^c^	–	38.3 ^d^	–
Thiamin (mg/d)	1.2 ± 0.0	1.1 (0.8, 1.5)	**0.5**	**0.7**	14.7	–	10.0 ^c^	–	19.4 ^d^	–
Riboflavin (mg/d)	1.1 ± 0.0	1.0 (0.7, 1.4)	**0.5**	**0.8**	21.8	–	13.6 ^c^	–	30.2 ^d^	–
Niacin (mg/d)	14.9 ± 0.3	13.3 (8.9, 18.7)	**6**	**9**	16.6	–	14.4	–	18.9	–
Vitamin B-6 (mg/d)	1.2 ± 0.0	1.0 (0.7, 1.5)	**0.5**	**0.8**	20.5	–	15.4 ^c^	–	25.7 ^d^	–
Folate (μg DFE/d)	238.0 ± 6.3	194.5 (116.3, 311.5)	**160**	**250**	52.9	–	42.0 ^c^	–	64.0 ^d^	–
Vitamin B-12 (μg/d)	2.2 ± 0.2	1.5 (0.6, 2.6)	**1**	**1.5**	44.6	–	39.7 ^a^	–	49.6 ^b^	–
Calcium (mg/d)	639.0 ± 14.3	553.2 (363.0, 830.9)	**800**	**1100**	81.3	–	76.1 ^c^	–	86.6 ^d^	–
Phosphorus (mg/d)	795.2 ± 13.4	747.3 (531.0, 1005.9)	**405**	**1055**	43.8	–	16.1 ^c^	–	72.2 ^d^	–
Magnesium (mg/d)	213.2 ± 3.6	197.8 (148.0, 260.9)	**110**	**200**	30.5	–	14.6 ^c^	–	46.7 ^d^	–
Vitamin D (μg/d)	1.4 ± 0.1	0.5 (0.1, 2.0)	**10**	**10**	99.4	–	99.2	–	99.5	–
Vitamin A (μg RAE/d)	530.8 ± 25.0	348.0 (177.0, 615.1)	**275**	**445 (B)-420 (G)**	69.5	–	60.3 ^c^	–	79.0 ^d^	–
Vitamin K (μg/d)	134.9 ± 7.6	71.3 (29.2, 151.7)	55	60	–	56.2	–	54.6	–	57.7
Iron (mg/d)	10.3 ± 0.2	9.0 (5.8, 13.2)	**4.1**	**5.9 (B)-5.7 (G)**	20.0	–	16.4 ^a^	–	23.6 ^b^	–
Zinc (mg/d)	6.6 ± 0.1	5.8 (4.1, 8.4)	**4**	**7**	41.1	–	26.7 ^c^	–	55.9 ^d^	–
Sodium (mg/d)	2050.9 ± 41.5	1875.3 (1209.0, 2586.6)	1000	1200	–	79.9	–	80.3	–	79.5
Potassium (mg/d)	2103.5 ± 38.4	1951.7 (1354.2, 2706.1)	2300	2500 (B)-2300 (G)	–	34.4	–	31.3	–	37.5

^1^DRIs in bold are EARs and those in Italic are AMDRs

^†^WHO upper limit for saturated fat (8% of energy intake) for children above 2 years of age (WHO, 2008)

^*^AHA recommendation for added sugars

^a^ and ^b^ indicate age differences in DRI compliance using chi-square test at *p* < 0.05

^c^ and d indicate age differences in DRI compliance using chi-square test at *p* < 0.001

The prevalence of children with carbohydrate intakes below EAR (g/d) was significantly higher among 4–8 years age group compared to 9–13 years group (10.3% compared to 5.8%), however, the proportion of children with carbohydrate intake (as %EI energy intake below AMDR) was found to be higher in the 9–13 years age (26.7% versus 33.9%), *p* < 0.05. On the other hand, the prevalence of children with protein intake below the EAR (g/d) was significantly higher among the older children (6.4% compared to 17.9%), *p* < 0.001. Moreover, an estimated 70% to 73% exceeded the AMDR for total fat (25% to 35% of energy), and 68.2% to 67.5% of the children exceeded the WHO upper level for saturated fat of 8% in the 4–8-years-old and 9–13-years-old children, respectively. In addition, 71% of 4–13-year-old children exceeded the AHA limits for added sugars (25 g per day), and a significantly higher proportion of children in the older age group (9–13 years-old-children) surpassed these limits compared to the younger ones (4–8 years) (75.6% vs. 66.4%, respectively, *p* < 0.05).

Results showed that the average intakes of vitamins and minerals were adequate amongst 4–13-year-old children, except for calcium and vitamin D, in which 81% and 99% of children’s intakes for these nutrients in the total sample were inadequate relative to the EAR. Other vitamins and minerals with high prevalence of inadequacy among 4–13-year-old-children were vitamin A (69.5%), folate (52.9%), vitamin B12 (44.6%), phosphorus (43.8%), zinc (41.1%), and vitamin C (31.7%). Mean potassium intake (2103.5 mg/d) was below the AIs (2300–2500 mg/d) for the total sample. As the requirements for the aforementioned nutrients increase as children get older (except for vitamin D), the percentage of children with inadequate intakes for these vitamins and minerals (relative to EAR) also increased significantly among the older age group (*p*-value < 0.05).

Energy and nutrient intakes were also examined by sex for 4 to 8 years old children in Table 3. For boys aged 4 to 8 years old, the percentages of energy from carbohydrate, total sugar, and protein fell within the AMDR, but percentages from total fat and saturated fat did not. An estimated 68.4% of boys exceeded the AMDR for total fat, and 67.9% exceeded the WHO upper level for saturated fat. Mean intakes of vitamin C, B vitamins, magnesium, and other micronutrients were adequate, except for vitamin D, calcium, vitamin A, and folate, for which 99%, 73.5%, 59.7%, and 41.8% of boys’ intakes in this age group for these nutrients were inadequate relative to the EAR, respectively. The average intakes of sodium from food exceeded the AI (1000 mg/d) in both boys’ and girls’ diets (1987.2 mg and 1774.1 mg) aged 4 to 8 years old. In addition, almost one-third of children 4–8-years old (57.2%, *n* = 223) exceeded the sodium levels (1500 mg/day), as per the most recent Chronic Disease Risk Reduction (CDRR) intake values (data not shown). On the other hand, the mean potassium intake was below the AI of 2300 mg/d for both boys and girls in this age group. Similarly, the percentage of energy from carbohydrate, total sugars, and protein fell within the AMDR for girls aged 4 to 8 years old, but percentages from total fat and saturated fat did not. An estimated 71% of girls exceeded the AMDR for total fat, and 68.6% exceeded the WHO upper level for saturated fat. Inadequacies were observed for vitamin D, calcium, vitamin A, and folate, in which 99.5%, 78.9%, 60.8%, and 42.3% of girls’ intakes in this age group for these nutrients were inadequate relative to the EAR, respectively. No significant differences in DRI compliance of any nutrient were observed between boys and girls in the 4 to 8 years age group.

**Table 3:** Macro- and micronutrient intakes for children aged 4 to 8 years (*n* = 390), by sex

Nutrient	DRI^1^	Boys (*n* = 196)					Girls (*n* = 194)			
	**AI/EAR/AMDR**	**Mean ± SE**	**Median (P25, P75)**	**% < EAR/AMDR**		**% > AI/AMDR**	**Mean ± SE**	**Median (P25, P75)**	**% < EAR/AMDR**	**% > AI/AMDR**
**Energy (kcal/d)**	–	1710.2 ± 49.1	1591.3 (1228.1, 2134.7)	–	–		1580.3 ± 49.4	1542.9 (1127.5, 1940.7)	–	–
**Macronutrients**										
Total Fat (g/d)	–	75.3 ± 2.7	68.0 (49.2, 96.2)	–	–		67.3 ± 2.4	63.7 (44.7, 84.9)	–	–
Saturated fat (g/d)	–	20.9 ± 1.0	18.2 (11.7, 26.6)	–	–		19.0 ± 0.8	16.1 (10.5, 25.6)	–	–
Carbohydrate (g/d)	**100**	213.0 ± 6.6	201.3 (149.0, 263.6)	8.7	–		201.9 ± 6.9	186.1 (139.0, 254.5)	11.9	–
Total Sugars (g/d)	**–**	74.6 ± 3.0	70.9 (43.8, 97.9)	–	–		73.2 ± 3.8	60.3 (39.7, 94.2)	–	–
Added Sugars (g/d)	25 ^a^	47.8 ± 2.8	39.6 (20.3, 68.0)	31.6	68.4		45.7 ± 3.2	38.6 (15.9, 60.3)	35.6	64.4
Dietary fiber (g/d)	25	13.3 ± 0.6	11.5 (7.6, 17.1)	–	7.7		13.1 ± 0.5	11.8 (7.4, 17.4)	–	6.7
Protein (g/d)	**0.76 g/Kg**	50.2 ± 1.7	46.0 (33.9, 61.6)	6.6	–		47.0 ± 1.7	43.6 (30.8, 56.0)	6.2	–
Total Fat (% of EI)	*25–35%*	38.9 ± 0.6	39.3 (33.2, 44.9)	6.6	68.4		37.9 ± 0.6	38.6 (34.0, 43.4)	7.7	71.1
Saturated fat (% of EI)	< *8%†*	10.8 ± 0.4	10.1 (7.1, 13.6)	–	67.9		10.7 ± 0.3	10.7 (7.3, 14.0)	–	68.6
Carbohydrate (% of EI)	*45–65%*	50.2 ± 0.7	50.3 (44.1, 55.7)	29.6	7.1		51.2 ± 0.7	50.5 (45.3, 55.9)	23.7	7.7
Total Sugars (% of EI)	*–*	18.1 ± 0.6	18.1 (11.7, 22.7)	–	–		18.4 ± 0.6	17.0 (12.1, 23.7)	–	–
Added Sugars (% of EI)	*–*	11.2 ± 0.5	10.5 (5.7, 15.7)	–	–		11.3 ± 0.6	10.3 (6.1, 15.1)	–	–
Protein (% of EI)	*10–30%*	12.0 ± 0.3	11.3 (9.1, 14.1)	34.2	1.0		12.2 ± 0.3	11.7 (9.2, 14.1)	31.4	0.5
**Micronutrients**										
Vitamin C (mg/d)	**22**	67.4 ± 4.7	47.7 (22.4, 88.2)	24.5	–		66.7 ± 6.0	44.5 (21.1, 83.3)	25.8	–
Thiamin (mg/d)	**0.5**	1.1 ± 0.0	1.1 (0.7, 1.4)	9.2	–		1.1 ± 0.0	1.0 (0.7, 1.3)	10.8	–
Riboflavin (mg/d)	**0.5**	1.1 ± 0.0	1.0 (0.7, 1.5)	11.7	–		1.0 ± 0.0	1.0 (0.6, 1.4)	15.5	–
Niacin (mg/d)	**6**	13.5 ± 0.5	12.5 (8.4, 17.5)	11.7	–		12.8 ± 0.6	11.6 (7.5, 15.5)	17.0	–
Vitamin B-6 (mg/d)	**0.5**	1.1 ± 0.0	1.0 (0.7, 1.4)	13.3	–		1.0 ± 0.0	0.9 (0.6, 1.3)	17.5	–
Folate (μg DFE/d)	**160**	229.9 ± 12.2	193.0 (98.5, 312.1)	41.8	–		219.4 ± 10.9	178.9 (103.8, 309.3)	42.3	–
Vitamin B-12 (μg/d)	**1**	2.0 ± 0.2	1.5 (0.6, 2.6)	38.8	–		1.8 ± 0.2	1.4 (0.5, 2.2)	40.7	–
Calcium (mg/d)	**800**	655.5 ± 27.8	557.5 (375.7, 848.9)	73.5	–		575.8 ± 26.7	491.5 (326.5, 743.6)	78.9	–
Phosphorus (mg/d)	**405**	767.1 ± 25.1	737.9 (507.3, 945.7)	14.8	–		716.8 ± 24.7	648.4 (492.1, 911.0)	17.5	–
Magnesium (mg/d)	**110**	202.3 ± 6.1	196.0 (137.5, 244.8)	12.8	–		196.3 ± 7.0	185.5 (130.9, 243.5)	16.5	–
Vitamin D (μg/d)	**10**	1.6 ± 0.2	0.8 (0.1, 2.5)	99.0	–		1.3 ± 0.1	0.5 (0.1, 1.9)	99.5	–
Vitamin A (μg RAE/d)	**275**	517.3 ± 40.7	323.7 (170.8, 635.8)	59.7	–		524.0 ± 51.4	344.4 (161.7, 612.0)	60.8	–
Vitamin K (μg/d)	55	108.1 ± 10.9	60.8 (25.4, 132.1)	–	51.5		105.2 ± 10.4	64.4 (30.0, 125.2)	–	57.7
Iron (mg/d)	**4.1**	10.3 ± 0.5	8.4 (5.9, 13.2)	13.3	–		9.2 ± 0.4	8.5 (5.0, 11.8)	19.6	–
Zinc (mg/d)	**4**	6.1 ± 0.2	5.7 (4.0, 7.9)	27	–		5.9 ± 0.2	5.3 (3.9, 7.4)	26.3	–
Sodium (mg/d)	1000	1987.2 ± 84.7	1750.4 (1175.7, 2510.0)	–	81.6		1774.1 ± 76.2	1588.8 (1069.7, 2216.2)	–	78.9
Potassium (mg/d)	2300	2004.3 ± 65.7	1903.7 (1397.3, 2567.3)	–	32.1		1915.9 ± 72.0	1861.9 (1203.4, 2465.1)	–	30.4

^1^DRIs in bold are EARs and those in Italic are AMDRs

^†^WHO upper limit for saturated fat (8% of energy intake) for children above 2 years of age (WHO, 2008)

^a^ AHA recommendation for added sugar

Differences in DRI compliance between boys and girls are tested using chi-square test. None has shown to be significantly different by sex

Table 4 presents the energy and nutrient intakes for 9–13-year-old children by sex. For children in this age group, around three-quarters of boys exceeded the AMDR for total fat whereas 70% among girls (*p*-value < 0.001). Significant differences were also observed between boys and girls in terms of AMDR compliance for protein as percent of energy intake (41.2% had intakes below AMDR in girls versus 29.4% in boys). Highest prevalence of vitamin and mineral inadequacies (relative to EAR) were observed for vitamin D, calcium, vitamin A, and folate in both sexes, with no significant differences between the two groups. For both boys and girls, average intakes of sodium from food exceeded the AI (2347.6 mg and 2083.0 mg, respectively) (Table 4) and 62.2% of 9–13 year-old-children (*n* = 381) exceeded sodium levels (1800 mg), as per the CDRR intake values (data not shown). In addition, mean potassium intakes were below the AI for this age group (2348.6 mg and 2136.3 mg, respectively). As presented in Table 4, a higher proportion of girls had inadequate intakes relative to EAR for thiamin, niacin, phosphorus, magnesium, and zinc compared to boys in the 9 to 13 years age group (*p*-value < 0.05).

**Table 4:** Macro- and micronutrient intakes for children aged 9 to 13 years (*n* = 381), by sex

Nutrient	DRI^1^	Boys (*n* = 204)				Girls (*n* = 177)			
		**Mean ± SE**	**Median (P25, P75)**	**% < EAR/AMDR**	**% > AI/AMDR**	**Mean ± SE**	**Median (P25, P75)**	**% < EAR/AMDR**	**% > AI/AMDR**
	**AI/EAR/AMDR**								
**Energy (kcal/d)**	–	2089.0 ± 58.1	2052.7 (1455.5, 2589.5)	–	–	1825.0 ± 53.7	1780.0 (1313.7, 2297.8)	–	–
**Macronutrients**									
Total Fat (g/d)	–	94.0 ± 3.1	89.7 (63.4, 122.8)	–	–	84.2 ± 3.1	78.1 (53.2, 112.3)	–	–
Saturated fat (g/d)	–	24.1 ± 1.0	22.0 (12.6, 33.1)	–	–	22.1 ± 1.0	20.5 (11.9, 29.0)	–	–
Carbohydrate (g/d)	**100**	253.1 ± 7.8	236.7 (168.6, 327.0)	5.4	–	219.0 ± 6.9	212.7 (148.4, 271.2)	6.2	–
Total Sugars (g/d)	–	84.7 ± 3.9	76.0 (41.7, 111.4)	–	–	77.4 ± 3.5	70.7 (44.6, 100.2)	–	–
Added Sugars (g/d)	25*	59.9 ± 3.3	48.9 (28.1, 80.3)	22.5	77.5	51.1 ± 2.9	43.2 (21.7, 75.5)	26.5	73.5
Dietary fiber (g/d)	31 (B)-26 (G)	16.1 ± 0.7	13.2 (9.0, 20.2)	–	9.3	14.0 ± 0.6	12.5 (9.7, 17.8)	–	6.8
Protein (g/d)	**0.76 g/Kg**	63.3 ± 2.1	61.2 (41.1, 78.8)	15.2	–	53.6 ± 2.0	49.2 (35.7, 67.6)	20.9	–
Total Fat (% of EI)	*25–35%*	39.9 ± 0.6	40..3 (35.2, 45.6)	8.8 ^a^	75.5 ^a^	40.3 ± 0.7	40.6 (34.1, 46.9)	5.1 ^b^	70.1 ^b^
Saturated fat (% of EI)	< *8%†*	10.1 ± 0.3	9.5 (7.1, 12.9)	–	65.7	10.7 ± 0.3	9.6 (7.1, 13.7)	–	69.5
Carbohydrate (% of EI)	*45–65%*	48.7 ± 0.7	48.4 (42.5, 54.4)	35.8	5.4	48.9 ± 0.7	49.2 (42.8, 54.7)	31.6	3.9
Total Sugars (% of EI)	*–*	16.2 ± 0.5	15.2 (10.4, 21.6)	–	–	17.1 ± 0.6	16.1 (11.2, 22.4)	–	–
Added Sugars (% of EI)	*–*	11.2 ± 0.5	9.7 (5.9, 16.1)	–	–	11.0 ± 0.5	9.6 (5.9, 15.6)	–	–
Protein (% of EI)	*10–30%*	12.5 ± 0.3	11.9 (9.4, 14.8)	29.4 ^a^	1.0 ^a^	12.1 ± 0.4	10.9 (8.8, 14.2)	41.2 ^b^	1.1 ^b^
**Micronutrients**									
Vitamin C (mg/d)	**39**	75.8 ± 5.2	53.3 (29.9, 102.7)	35.8	–	71.9 ± 5.2	47.1 (21.5, 101.5)	41.2	–
Thiamin (mg/d)	**0.7**	1.4 ± 0.0	1.3 (0.9, 1.7)	15.7 ^a^	–	1.1 ± 0.0	1.0 (0.7, 1.4)	23.7 ^b^	–
Riboflavin (mg/d)	**0.8**	1.2 ± 0.1	1.1 (0.8, 1.5)	28.4	–	1.1 ± 0.0	0.9 (0.7, 1.3)	32.2	–
Niacin (mg/d)	**9**	18.4 ± 0.7	17.0 (10.9, 21.9)	14.2 ^a^	–	14.8 ± 0.7	13.1 (9.2, 17.7)	24.3 ^b^	–
Vitamin B-6 (mg/d)	**0.8**	1.4 ± 0.1	1.3 (0.8, 1.8)	22.1	–	1.2 ± 0.1	1.1 (0.7, 1.4)	29.9	–
Folate (μg DFE/d)	**250**	264.7 ± 14.4	206.6 (139.8, 326.3)	62.3	–	236.4 ± 12.1	197.6 (116.6, 310.6)	66.1	–
Vitamin B-12 (μg/d)	**1.5**	3.0 ± 0.6	1.7 (0.6, 3.1)	45.1	–	2.0 ± 0.2	1.4 (0.5, 2.4)	54.8	–
Calcium (mg/d)	**1100**	697.8 ± 28.2	634.6 (392.3, 906.6)	86.8	–	622.4 ± 31.1	521.0 (335.9, 779.7)	86.4	–
Phosphorus (mg/d)	**1055**	909.9 ± 28.1	849.1 (622.9, 1168.3)	66.7 ^a^	–	780.3 ± 26.9	719.0 (521.6, 978.9)	78.5 ^b^	–
Magnesium (mg/d)	**200**	238.4 ± 7.6	220.7 (162.0, 303.0)	40.7 ^a^	–	214.8 ± 7.9	195.0 (148.2, 256.3)	53.7 ^b^	–
Vitamin D (μg/d)	**10**	1.6 ± 0.2	0.5 (0.1, 2.2)	99.0	–	1.1 ± 0.1	0.3 (0.1, 1.2)	100	–
Vitamin A (μg RAE/d)	**445 (B)-420 (G)**	531.2 ± 48.5	373.9 (189.4, 638.4)	77.9	–	552.8 ± 59.6	321.2 (172.9, 577.9)	80.2	–
Vitamin K (μg/d)	60	164.7 ± 16.8	85.0 (38.1, 183.1)	–	62.8 ^a^	162.8 ± 20.6	73.7 (27.4, 181.2)	–	52.0 ^b^
Iron (mg/d)	**5.9 (B)-5.7 (G)**	11.5 ± 0.5	10.4 (6.7, 15.1)	22.1	–	10.0 ± 0.5	8.2 (5.7, 12.9)	25.4	–
Zinc (mg/d)	**7**	7.7 ± 0.3	7.3 (4.5, 10.2)	48.5 ^a^	–	6.6 ± 0.3	5.7 (3.8, 8.2)	64.4 ^b^	–
Sodium (mg/d)	1200	2347.6 ± 84.5	2221.0 (1441.6, 3046.6)	–	80.9	2083.0 ± 80.8	2056.2 (1271.3, 2587.8)	–	78.0
Potassium (mg/d)	2500 (B)-2300 (G)	2348.6 ± 81.6	2085.7 (1465.5, 3109.7)	–	39.2	2136.3 ± 84.3	1961.7 (1392.3, 2563.4)	–	35.6

^1^DRIs in bold are EARs and those in Italic are AMDRs

^†^WHO upper limit for saturated fat (8% of energy intake) for children above 2 years of age (WHO, 2008)

^*^AHA recommendation for added sugar

^a^ and ^b^ indicate sex differences in DRI compliance using chi-square test at *p* < 0.05

c and d indicate sex differences in DRI compliance using chi-square test at *p* < 0.001

## Discussion

The present study aimed to characterize the dietary intakes of Lebanese school-aged children (4–13-years-old) and to assess their adherence to evidence-base nutrition guidelines and dietary recommendations. Our results showed that more than two-thirds of 4–13-year-old Lebanese children had high intakes of total fat, saturated fats and added sugars, exceeding the respective dietary recommendations. Sex-based differences in food consumption patterns were detected, particularly among younger children (4–8-year-olds). Micronutrient inadequacies were also noted for key vitamins and minerals among the study sample and the risk of inadequacy was higher among the older age group.

Overall, fat and saturated fat intakes were found to be high among Lebanese school-aged children in the present study (39.2% EI from fat and 11% EI from saturated fats) with an estimated 68% and 71% of 4–13 year old children exceeding the AMDR for total fat and saturated fats, respectively. Similar results were observed in previous studies conducted amongst Lebanese school-aged children with fat intakes ranging between 35.8% and 39.7% EI, and more than half of the children exceeding the upper levels for saturated fat intake [18]. Such findings are also comparable to those reported in Iran whereby children had intakes of saturated fat above the recommendations for boys and girls (65.7% and 58.8% of EI from saturated fat, respectively) [52]. The proportions of children exceeding intakes of total fat in our study sample remained higher than those reported among school-aged children in other countries in the region, including Kuwait and UAE (36.3–40.8% of 4-18-year-olds and 5–20% of 6-18-year-olds exceeded total fat AMDR, respectively) [53, 54]. When compared to other studies conducted using the KNHS protocol, the proportions of Lebanese 4-13-year-old children exceeding the saturated fat intake recommendations in the present study were similar to those reported in Mexico (68% and 83%) and in China (60% and 55%), for boys and girls, respectively [55, 56]. These results are alarming, because high intakes of dietary fats, particularly saturated fat, are risk factors for overweight and obesity and early development of non-communicable chronic diseases, including hypertension and type II diabetes, among children [57–59].

Our results also showed that the majority of 4–13-year-old children exceeded the AHA limits for added sugars (25 g per day), and a significantly higher proportion of children in the older age group (9–13 years-old-children) surpassed these limits compared to the younger ones (4–8 years) (75.6% vs. 66.4%, respectively). In parallel, sweets, sweetened beverages, desserts were found to be the main contributors of energy intake among 4–13-year-old Lebanese children. The high intake of added sugars among children and adolescents in the present study may thus be explained by the high intake of sweets, sweetened beverages, and desserts (e.g. chocolates, candies and sugared soft drinks), which was accompanied by an inadequate intake of calcium-rich drinks, such as milk and milk products, particularly among the older age group. Authors have previously documented the higher contribution of sugar-sweetened beverages, such as regular sodas and fruit drinks, to total daily water and energy intake of Lebanese children and adolescents, whereas milk intake contributed to less than 3% of children’s daily energy intake [60]. Sugar sweetened beverages are in fact the leading sources of added sugars in the diet of children worldwide and can contribute to their overall energy density and to poor health outcomes [61]. Furthermore, findings derived from randomized clinical trials and epidemiologic studies showed that individuals who consume high amounts of added sugars, especially sugar-sweetened beverages, have a higher risk of obesity [62, 63], type 2 diabetes [62, 64], dyslipidemias [65, 66], hypertension [67, 68], cardiovascular diseases [64, 66] and dental caries [69, 70].

The intake level of Lebanese children from different food groups were compared to the dietary recommendations set by AHA/AAP. Results showed that a high proportion of 4–13-year-old children were not adhering to the recommended intakes for several food groups, including lean meat/beans, milk/dairy, fruits, and vegetables. In addition, 9–13-year-old Lebanese children were significantly less adherent to the recommendations for milk and dairy as well as vegetables compared to their younger counterparts (4–8-year-old children). Such unhealthy consumption patterns and practices may be explained by the nutrition transition that has been witnessed over the last few decades among the Lebanese population, including children and adolescents, with a higher consumption of low-nutrient, energy-dense foods and beverages coupled with increased sedentary behaviors [26]. As the nutrition transition is unfolding, the traditional Mediterranean diet, characterized by widespread consumption of fruits, vegetables, whole-grain cereals, legumes, nuts and seeds, is progressively eroding and being replaced with more Westernized-like dietary patterns that are more convenient, appealing and heavily promoted among adolescents [29]. Our results were also consistent with previous studies conducted among youth in other Middle Eastern countries that showed the consumption of nutrient-dense foods, such as fruits, vegetables, and milk, to be lower than the recommendations amongst 6–18-year-old Emirati children [53, 71] and reflecting overall poor diet quality among 13- to 18-year-old adolescents in Saudi Arabia [72]. The nutrition transition has been linked to the escalating burden of overweight, obesity, and non-communicable diseases in EMR countries throughout the lifecycle [73–75].

Additionally, it is important to note that a high proportion of Lebanese children (4-13 years) in the present study had inadequate intakes (relative to EAR) of essential vitamins and minerals including vitamin D (99.4%), calcium (81.3%), vitamin A (69.5%), folate (52.9%), and zinc (41%). Yet, the proportion of children not meeting the DRI requirements for micronutrients was found to be significantly higher among the older age children (9–13-year-old) compared to younger ones in the present study. A recent review on the nutrition situation of children in the EMR showed similar inadequate intakes of iron, calcium, zinc, folic acid, vitamin A, and vitamin D amongst school-aged children in Lebanon, UAE, and KSA [18]. Previous studies conducted in Lebanon more than a decade ago showed that 73% to 88% and 84% to 95% of school-aged children were not meeting two-thirds of the recommended dietary allowances for calcium and vitamin D, respectively [76, 77]. Similarly, in the UAE, researchers found that the majority (> 80%) of 6–13-year-old children were not meeting the respective EAR levels for vitamins A, D, E, and of calcium [53]. Given the increased nutrient requirements for children during puberty and its accompanying growth spurt, such inadequacies can have detrimental effects on adolescents’ physical health such as delaying their sexual development and slowing their linear growth [12]. Micronutrient inadequacies during childhood and adolescence can also affect cognitive function and inhibit academic performance [13, 15]. Inadequate intakes of micronutrients along with the consumption of energy-dense, nutrient-poor diets that are high in fat and sugar and low in fiber can also increase the risk for obesity and non-communicable diseases among adolescents and throughout their life cycle [78–81].

In terms of macro and micronutrients, sex-based differences were mostly noted amongst the 9–13-year-old children with a significantly higher proportion of girls compared to boys not meeting the DRIs for protein intake and key micronutrients, including thiamin, niacin, phosphorus, magnesium and zinc. These discrepancies may be attributed to the slightly lower consumption of animal-based protein sources such as meat and other protein sources amongst girls compared to boys in the older age group. Differences in nutrients intake may be also attributed to social norms and differences in intra-household food distribution and allocation that favor men and boys over women and girls, in many Middle Eastern countries, including Lebanon [28, 82]. Using the 2008–2009 national survey, authors previously showed sex discrepancies in the dietary intake of Lebanese children and adults across the lifespan with females having lower micronutrient intakes and being at higher risk of micronutrient inadequacies, including calcium, iron, zinc, and vitamin B12, compared to their male counterparts [28]. Significant gender differences were also observed among Kuwaiti children with lower intakes for vitamin B12, zinc, calcium and phosphorus documented among girls compared to boys [54]. Importantly, micronutrient inadequacies amongst youth, particularly adolescent girls, can have adverse effects on their physiological performance and may contribute to poor pregnancy outcomes later in life [17, 83]. Research shows that adolescent girls need proper nutrition to improve their health and that of their future offspring and families, thus breaking the intergenerational cycle of malnutrition [84, 85].

With almost 44% of children in the study sample assessed to be at risk of overweight or overweight status and with the documented nutrient intakes of school-aged children in Lebanon reflecting suboptimal nutrition, our findings further validate previous research that recognized the multiple burden of childhood malnutrition, with overnutrition occurring simultaneously along with multiple micronutrient deficiencies, in LMICs including those in the EMR [4, 18]. Gender-based disparities in dietary have been also documented in the present study and highlight the need to further explore cultural and social norms that continue to favor boys and men over women in many Arab countries within the region [23, 28, 86, 87]. Such practices may further contribute to the poor nutritional status of children, primarily adolescent girls, on the short and long-term.

### Strengths and limitations

Data for the present study was retrieved from a nationally representative survey of Lebanese households with school-aged children that followed a rigorous data collection protocol to ensure data accuracy and minimize errors [31]. Dietary intake was also collected by trained nutritionists and dietitians who received extensive training prior to data collection to reduce social desirability and recall biases. Nevertheless, one of the limitations of the study is the reliance on a single 24-h recall from each child participant to calculate nutrient intake. As such, we are unable to measure the degree of intra-individual variation in this population; and therefore, it is unknown how representative the reported food consumption is of usual intakes of children. Nevertheless, the research team followed the USDA multiple pass recall method that provides ample opportunities to identify foods and specific details about the foods consumed during the recall period, thus limiting the degree of bias [88]. In addition, the research team exerted every attempt to minimize factors leading to unusual eating patterns by avoiding data collection during religious fasting months, festive holidays, and summer months [89]. Although dietary data is self-reported and underreporting may have occurred, past research has found that 24- hour diet recalls show substantially smaller biases than food frequency questionnaires [90]. Other limitations include incomplete data on saturated fatty acids content for several local food items within our food composition tables and the USDA database; in addition, the present study lacked data on the use of vitamin and mineral supplements among Lebanese children. Thus, future studies need to take into consideration supplement use amongst children and its contribution to usual nutrient intake and adequacy. In addition, qualitative studies that can complement existing quantitative analyses of the dietary intake and behaviors of children and youth are needed in various LMIC settings, including Lebanon. These studies can help explore the perceptions and acceptability of children and youth towards specific dietary behaviors and patterns, including the Mediterranean diet and its specific food components, and identify potential barriers of consumption to address in future public health nutrition programs and policies.

## Conclusion

In conclusion, the present study shows a high intake of fats, saturated fats, and added sugars amongst Lebanese school-aged children. In addition, a high proportion of children are not meeting the dietary recommendations for food groups, including lean meat and beans, fruits, vegetables as well as milk and dairy. Results also highlight the suboptimal intakes of key vitamins and minerals, including vitamin D, vitamin A, vitamin B12, folate, calcium, zinc, and folic acid. Age and sex-based differences in dietary intake and inadequacies were also documented in this study with older children and females being at higher risk of inadequacies.

Educational and public health interventions are needed to promote healthier diets among children and to prevent the risk of excessive weight gain along with micronutrient deficiencies during this critical phase. Such interventions need to be based on culturally specific, science-driven food-based dietary guidelines (FBDGs) and recommendations that remain lacking in many countries in the MENA region, including Lebanon. Thus, concerted efforts are required to devise FBDGs and implementation plans that are coherently integrated into the national food, agriculture, health policies and programs and can engage a wide range of stakeholders from the government, non-governmental, private, academic and media sectors. Such public health interventions ought to be also tailored to the needs and preferences of school-aged children while addressing cultural barriers that may contribute to age- and sex-based disparities in dietary intake. In addition, special attention should be given to adolescence, a unique period in which nutritional interventions can help provide the education and proper nutrition to promote optimal health and reduce the risk of malnutrition and chronic diseases in future generations.

## Supplementary Information

**Additional file 1: **Mean intake for different food groups amongst Lebanese children aged 4 to 13 years per capita, by age groups (aggregated mixed dishes) 

**Additional file 2: **Median intake for different food groups amongst Lebanese children aged 4 to 13 years per capita, by age groups (aggregated mixed dishes) 

**Additional file 3: **American Heart Association recommended servings per food group 

## Data Availability

The authors confirm that the data supporting the findings of this study are available within the manuscript and its supplementary material. Raw data that support the findings of this study are available from the corresponding authors, upon reasonable request.
